# High-Quality Draft Genome Sequence of the Siderophilic and Thermophilic *Leptolyngbyaceae* Cyanobacterium JSC-12

**DOI:** 10.1128/MRA.00495-21

**Published:** 2021-06-24

**Authors:** Igor Brown, Susannah G. Tringe, Natalia Ivanova, Lynne Goodwin, Nicole Shapiro, Jaime Alcorta, Donald Pan, Andrei Chistoserdov, Svetlana Sarkisova, Tanja Woyke

**Affiliations:** a Jacobs Engineering/NASA Johnson Space Center, Houston, Texas, USA; b U.S. Department of Energy Joint Genome Institute, Berkeley, California, USA; c Los Alamos National Laboratory, Los Alamos, New Mexico, USA; d Department of Molecular Genetics and Microbiology, Biological Sciences Faculty, Pontifical Catholic University of Chile, Santiago, Chile; e Department of Ecology and Environmental Studies, The Water School, Florida Gulf Coast University, Fort Myers, Florida, USA; f Department of Biology, University of Louisiana at Lafayette, Lafayette, Louisiana, USA; University of Delaware

## Abstract

The siderophilic, thermophilic *Leptolyngbyaceae* cyanobacterium JSC-12 was isolated from a microbial mat in an iron-depositing hot spring. Here, we report the high-quality draft genome sequence of JSC-12, which may help elucidate the mechanisms of resistance to extreme iron concentrations in siderophilic cyanobacteria and lead to new remediation biotechnologies.

## ANNOUNCEMENT

Despite the extreme oxidative stress induced by high Fe(II) concentrations (>100 μM) (1), contemporary circumneutral iron-depositing hot springs are densely populated with cyanobacteria (2–4). Here, we report the high-quality draft genome sequence of the cyanobacterium JSC-12, isolated from a phototrophic mat formed on iron deposits in Chocolate Pots Hot Springs, Yellowstone National Park, WY, USA (5). A sample was aseptically collected from a surface mat located ∼1 m downstream from the main mound (5), where the water was 40°C, pH 6.48, and contained 2.8 μM ferrous and 4.2 μM ferric iron. From this, we obtained a unialgal isolate of JSC-12 following references 2 and 4, using DH growth medium (6) supplemented with 0.04 mM FeCl_3_·6H_2_O. JSC-12 is a filamentous cyanobacterium with narrow trichomes (0.8 to 1.8 μm wide), resembling the morphological features of *Leptolyngbya* (7). Strain JSC-12 showed thermophilic and siderophilic behavior with an optimal growth temperature of 45°C and an Fe(III) concentration of 0.4 to 0.8 mM (3).

For genomic DNA isolation, JSC-12 was cultivated at 40°C under permanent white light (17 μE/m^2^/s) in 1-liter flasks containing 0.25 liter of DH medium supplemented with 0.04 mM FeCl_3_·6H_2_O on a rotating shaker (18 rpm). The genomic DNA was purified using the UltraClean microbial DNA isolation kit (MoBio Laboratories, Carlsbad, CA, USA). The genome was sequenced from an Illumina regular fragment library using the KAPA-Illumina library creation kit (Kapa Biosystems) (96,333,258 reads and 7,321.3 Mb) and a paired-end 454 library (220,108 reads and 58.1 Mb) according to Peng et al. (8). Prior to assembly, the reads were filtered to remove low-quality reads, Escherichia coli strains, 454 adapters, and known contaminants using tools from the 454 assembler suite (Roche), including runMapping and runAssembly. The remaining data were assembled using a combination of Newbler v. 2.3-PreRelease-6/30/2009, VELVET v. 1.0.13 (9), and parallel Phrap v. 1.080812. The final high-quality draft assembly contained 20 contigs in 1 scaffold.

Annotation was performed by Oak Ridge National Laboratory using Prodigal v. 1.4 (10), followed by manual curation using the U.S. Department of Energy Joint Genome Institute’s GenePRIMP pipeline (11). For all computational steps, default parameters were used except where otherwise noted. The JSC-12 genome has a 5,530,491-bp genome with a GC content of 47.5; it contains 5,080 genes, 2 rRNA operons, and 44 tRNA genes. Phylogenetic analyses showed that JSC-12 is associated with *Leptolyngbya*, but the similarity in 16S rRNA (∼92%) and genomic amino acid identity (62 to 65%), along with genomic taxonomy from GTDB (12), suggest that it belongs to a potential new genus in the *Leptolyngbyaceae* family (13, 14).

Prediction of biosynthetic gene clusters revealed that the JSC-12 genome lacks the pathway for synthesizing bacterioferritin, the classical iron storage protein. However, the gene *rbr*, encoding rubrerythrin (15), was present. A putative *feo*ABC operon (16) may be involved in the acquisition of Fe(II) through the cytoplasmic membrane for sequestration and detoxification within ferritin (*ftn*), which is likely cotranscribed with *feoABC*.

### Data availability.

The JSC-12 genome can be found under the GenBank Nucleotide accession numbers CM001633.1, AJUB00000000.1, and GCF_000309945.1. The raw reads are available under the SRA accession numbers SRX1954729 and SRX10660385.
